# Binding of the Heterogeneous Ribonucleoprotein K (hnRNP K) to the Epstein-Barr Virus Nuclear Antigen 2 (EBNA2) Enhances Viral LMP2A Expression

**DOI:** 10.1371/journal.pone.0042106

**Published:** 2012-08-03

**Authors:** Henrik Gross, Christine Hennard, Ilias Masouris, Christian Cassel, Stephanie Barth, Ute Stober-Grässer, Alfredo Mamiani, Bodo Moritz, Dirk Ostareck, Antje Ostareck-Lederer, Nils Neuenkirchen, Utz Fischer, Wen Deng, Heinrich Leonhardt, Elfriede Noessner, Elisabeth Kremmer, Friedrich A. Grässer

**Affiliations:** 1 Institute of Virology, Saarland University Medical School, Homburg/Saar, Germany; 2 Institute of Molecular Immunology, Helmholtz Zentrum München, German Research Center for Environmental Health, Munich, Germany; 3 Institute of Biochemistry and Biotechnology, Martin-Luther-University Halle-Wittenberg, Halle (Saale), Germany; 4 Experimental Research Unit, Department of Intensive Care and Intermediate Care, University Hospital Aachen, RWTH Aachen University, Aachen, Germany; 5 Department of Biochemistry, Biocenter of the University of Würzburg, Würzburg, Germany; 6 Department of Biology, Center for Integrated Protein Science Munich, Ludwig Maximilians University Munich, Planegg-Martinsried, Germany; University of Hong Kong, Hong Kong

## Abstract

The Epstein-Barr Virus (EBV) -encoded EBNA2 protein, which is essential for the *in vitro* transformation of B-lymphocytes, interferes with cellular processes by binding to proteins via conserved sequence motifs. Its Arginine-Glycine (RG) repeat element contains either symmetrically or asymmetrically di-methylated arginine residues (SDMA and ADMA, respectively). EBNA2 binds via its SDMA-modified RG-repeat to the survival motor neurons protein (SMN) and via the ADMA-RG-repeat to the NP9 protein of the human endogenous retrovirus K (HERV-K (HML-2) Type 1). The hypothesis of this work was that the methylated RG-repeat mimics an epitope shared with cellular proteins that is used for interaction with target structures. With monoclonal antibodies against the modified RG-repeat, we indeed identified cellular homologues that apparently have the same surface structure as methylated EBNA2. With the SDMA-specific antibodies, we precipitated the Sm protein D3 (SmD3) which, like EBNA2, binds via its SDMA-modified RG-repeat to SMN. With the ADMA-specific antibodies, we precipitated the heterogeneous ribonucleoprotein K (hnRNP K). Specific binding of the ADMA- antibody to hnRNP K was demonstrated using *E. coli* expressed/ADMA-methylated hnRNP K. In addition, we show that EBNA2 and hnRNP K form a complex in EBV- infected B-cells. Finally, hnRNP K, when co-expressed with EBNA2, strongly enhances viral latent membrane protein 2A (LMP2A) expression by an unknown mechanism as we did not detect a direct association of hnRNP K with DNA-bound EBNA2 in gel shift experiments. Our data support the notion that the methylated surface of EBNA2 mimics the surface structure of cellular proteins to interfere with or co-opt their functional properties.

## Introduction

The Epstein-Barr virus (EBV) is associated with various human malignancies [1] and growth-transforms primary human B-lymphocytes which are the *in vitro* correlate of EBV-associated post-transplant lymphoproliferative disease (PTLD) (for review, see [2]). In EBV-transformed lymphocytes, 11 so-called latent genes are expressed. Of these, only the nuclear antigens EBNA1, -2, -3a, -3c and the latent membrane protein LMP1 are necessary for transformation (reviewed in [3]).

**Figure 1:**
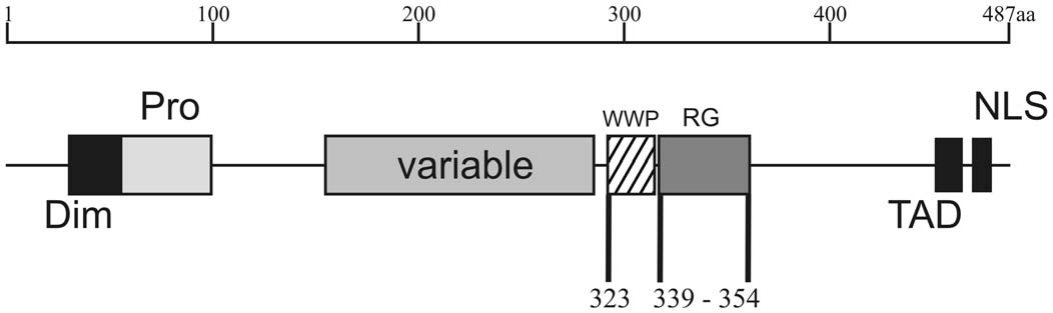
Schematic representation of the Epstein-Barr virus nuclear antigen 2 (EBNA2). EBNA2 of the standard B95.8 strain (accession number: AJ507799) of EBV consists of 487 amino acids (aa) present in an A-type virus. The N-terminal dimerization domain (“Dim”) is located next to a poly-Proline stretch (“Pro”). The variable region (“variable”) differs between the A-type viruses and B-type viruses. B-type viruses have a reduced *in vitro* transformation potential. The binding site for RBPjκ (“WWP”) is located around a Trp-Trp-Pro motif at aa 323–325. The adjacent Arginine-Glycine repeat (“RG”) between aa 339–354 confers binding to the survival of motor neurons (SMN) protein and represents the second nuclear localization signal (“NLS”) in addition to the canonical NLS found at the extreme C-terminus between aa 468–487. The C-terminal acidic transactivation domain (“TAD”) between aa 424–468 interacts with various basal transcription factors.

EBNA2 is a multifunctional transcriptional activator that does not bind directly to DNA but is tethered to promoter elements by interacting with DNA-bound transcription factors. For example, it associates through a Trp-Trp-Pro (“WWP_325_”) motif at position 323–325 (see Figure 1) with the DNA-bound repressor RBPjκ [4], [5], [6]. EBNA2 is the viral functional homologue to the cellular transmembrane receptor Notch which also activates gene expression via RBPjκ (reviewed in [7]). Binding of EBNA2 or Notch converts the repressor RBPjκ to the transcriptionally active form. Figure 1 shows a schematic representation of EBNA2. A virus encoding an EBNA2 protein with a mutation in the WWP-motif is unable to immortalise B-lymphocytes and does not activate the viral oncogene LMP1 [8]. In addition to RBPjκ, EBNA2 binds to a variety of basal transcription factors [2] and forms complexes with proteins involved in RNA metabolism like the DEAD-box protein DDX20 (DP103/Gemin3) [9] or the survival of motor neurons (SMN) protein [10], [11]. The binding of EBNA2 to a variety of other host proteins is reflected by its presence in high molecular weight complexes of different composition [12], [13], [14]. Adjacent to the WWP-motif, EBNA2 contains an Arginine-Glycine (RG-) repeat element at aa 339–354 with methylated arginine residues [10], [15]. The deletion of the RG-repeat results in a five-fold higher ability of EBNA2 to stimulate LMP1 expression, but a recombinant virus featuring this deletion in EBNA2 has a reduced transforming activity and needs an extended time span to induce outgrowth of transformed cell clones [16]. The EBNA2A protein from type A isolates was originally shown to confer a higher transforming capacity than EBNA2B derived from type B isolates of EBV [17]. Recently, it was demonstrated that the RG- repeat, among other C-terminal sequences, is important to confer the higher transforming activity of EBNA2A vs. EBNA2B [18].

Methylation is a post-translational modification that affects protein-protein interactions [19]. Methylation at arginine residues [20] may lead to three known forms in higher eukaryotes: ω-N^G^ MonoMethyl-arginine (MMA), ω-N^G^,N^G^-Asymmetric DiMethyl-arginine (ADMA) and ω -N^G^,N’^G^-Symmetric DiMethyl-arginine (SDMA). The methylation is carried out by two types of Protein-Arginine-Methyl-Transferases (PRMTs): Type I enzymes (PRMT1, 2, 3, 4, 6 and -8) catalyse the formation of ADMA whereas type II enzymes (PRMT5, -7 and -9) account for the formation of SDMA ([21], [22], [23].

**Figure 2:**
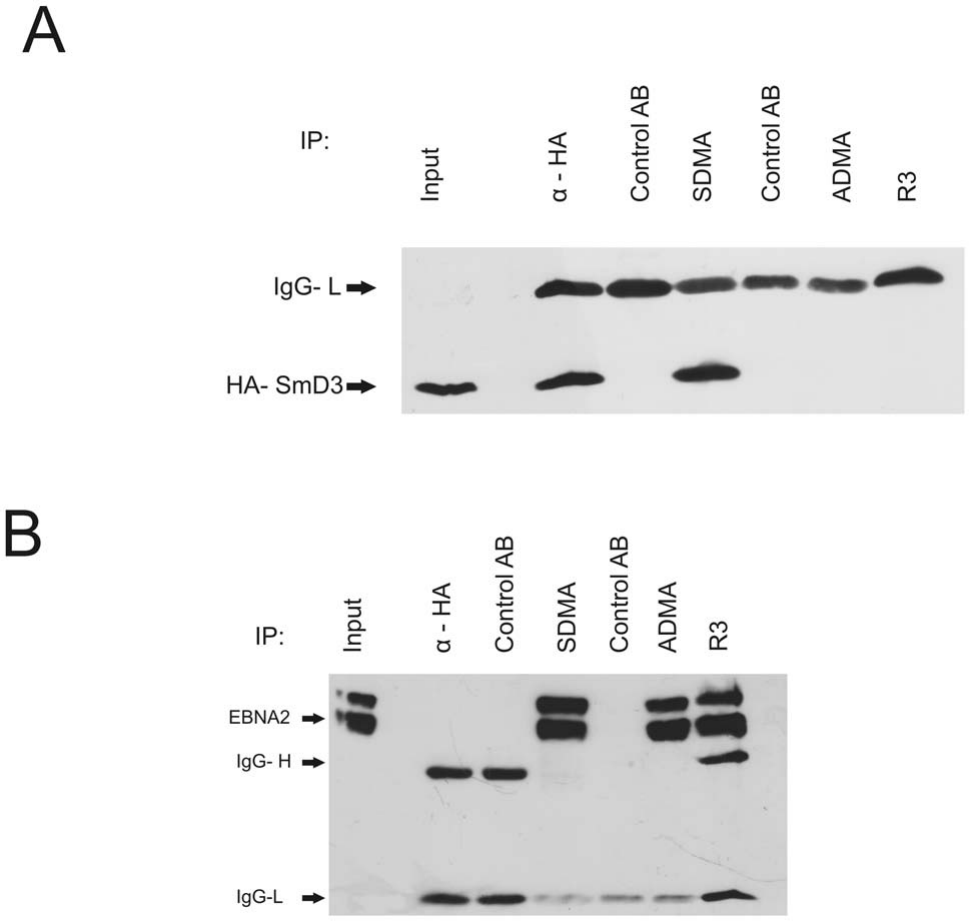
SmD3 is precipitated by the SDMA- EBNA2 specific antibody. (A) Monoclonal antibodies (mAbs) directed against the SDMA- and ADMA- containing Arginine-Glycine (RG)-repeat of EBNA2 were tested by precipitation using extracts of HEK 293-T cells expressing EBNA2-wt and HA- SmD3. For each antibody, an appropriate isotype control was tested in parallel to exclude unspecific binding to the protein G Sepharose used for precipitation. Precipitated HA- SmD3 protein was visualised using the HA -specific mAb 3F10. The position of HA- SmD3 is indicated by an arrow. (B) Immunoprecipitation of EBNA2 from transiently transfected cells. HEK 293-T cells expressing EBNA2-wt and HA- SmD3 were precipitated with monoclonal antibodies directed against the SDMA- and ADMA- containing Arginine-Glycine (RG)-repeat of EBNA2 using appropriate isotype control antibodies. Precipitated EBNA2 protein was visualised using the EBNA2 mAb R3. The position of EBNA2 is indicated by an arrow.

**Table 1:** Proteins precipitated by the EBNA2- **SDMA** antibody.

Protein	Size	Accession number	Methylationstatus
Pre-mRNA processing splicing factor 8	∼200 kDa	Q6P2Q9	unknown
U5 small nuclear ribonucleoprotein 200 kDa helicase	∼200 kDa	O75643	unknown
Gem-associated protein 5 (gemin 5)	∼150 kDa	Q8TEQ6	unknown
U1 snRNP 70 K	∼55 kDa	P08621	sDMA
SmD3	∼14 kDa	P62318	sDMA
**Proteins precipitated by the EBNA2- ADMA antibody**			
**Protein**	**Size**	**Accession number**	**Methylationstatus**
ATP dependent RNA helicase	∼140 kDa	Q08211	aDMA
Caprin-1	∼80 kDa	Q14444	unknown
Ras-GTPase-activating protein (SH3-domain-binding protein variant)	∼55 kDa	Q53HH4	unknown
hnRNP K	∼55 kDa	Q5T6W5	aDMA

hnRNP K was originally detected as a polycytidylic acid binding protein purified from heterogeneous nuclear ribonucleoprotein particles [24]. Later on, it was found that hnRNP K is involved in various cellular processes such as chromatin reorganisation, mRNA translation, transcriptional regulation, splicing, RNA shuttling and cell survival (for review, see [25], [26]). Recently, it was proposed that hnRNP K activates the VEGF-A promoter by binding to unwound superhelical single stranded C-rich sequences upstream of the transcription start site and to support association of transcription initiation factors [27]. hnRNP K is composed of modular regions that confer binding both to RNA or DNA as well as protein-protein interaction domains [28]. It binds to tyrosine kinases like c- Src and Lck as well as transcription factors such as C/EBP. The interaction with c-Src and its activation by hnRNP K is modulated by asymmetric dimethylation of five arginine residues catalysed by PRMT1 [29]. Reduced PRMT1 expression in induced erythroid maturation of human K562 cells leads to a decreased methylation of newly synthesised hnRNP K. This correlates with hnRNP K tyrosine phosphorylation and activation of the human reticulocyte 15-lipoxygenase (r15-LOX) mRNA translation, which is inhibited by hnRNP K in early erythroid maturation [30], [31].

**Figure 3:**
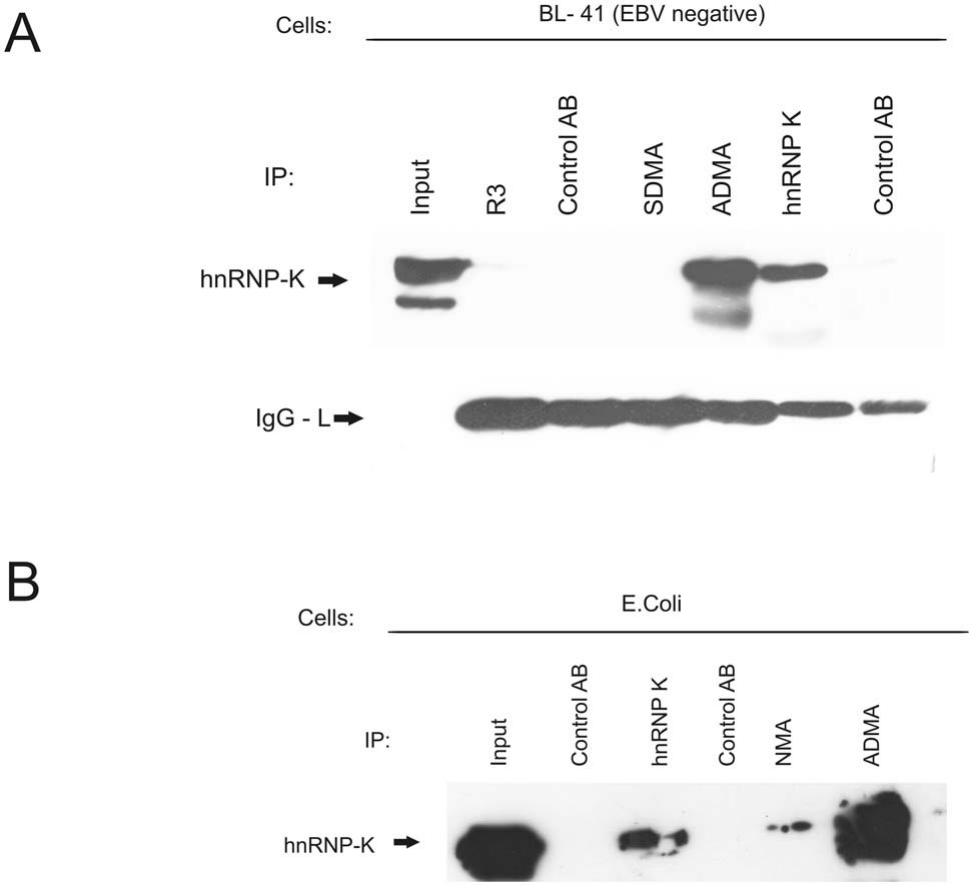
hnRNP K is precipitated by the ADMA- specific antibody. (A) Immunoprecipitation of hnRNP K from BL- 41 cells. EBV negative BL- 41 cells were precipitated with monoclonal antibodies directed against the SDMA- and ADMA- containing Arginine-Glycine (RG)-repeat of EBNA2 and an hnRNP K specific antibody using appropriate isotype control antibodies. The EBNA2 specific mAb R3 served as a negative control. The position of hnRNP K is indicated by an arrow. (B) ADMA- modified hnRNP K is precipitated by the ADMA- specific antibody. Soluble extract containing ADMA- hnRNP K methylated in *E. coli* with the type I methyltransferase PRMT1 was subjected to immunoprecipitation using either a hnRNP K- specific mAb, the ADMA- specific mAb and the NMA- specific mAb. Precipitated hnRNP K was visualised using the hnRNP K mAb.

We have previously developed monoclonal antibodies against the methylated RG-repeat of EBNA2 and found that it contains either SDMA or ADMA residues but does not exist in non-methylated (NMA) form [15]. SDMA-modified EBNA2 (SDMA-EBNA) forms a complex with the SMN protein [10], while ADMA-modified EBNA2 (ADMA-EBNA2) preferentially binds to the NP9 protein encoded by the human endogenous retrovirus HERV-K (HML-2) Type 1 [32]. It is known that SMN binds only to SDMA-modified proteins [33]; [34], for example to the SDMA-modified SmD3 protein which in turn is part of the SMN complex [35]. We therefore reasoned that the methylated surface of EBNA2 at the RG-repeat resembles cellular proteins engaging in similar interactions and that the antibodies should recognize these conserved epitopes. These cellular proteins in turn should be able to interact with proteins bound to EBNA2. Here we show that the immunoprecipitation of cellular proteins using the SDMA-specific antibodies indeed yields SmD3. Likewise, the ADMA-antibodies precipitated, among other proteins, hnRNP K. Further analysis demonstrated that EBNA2 does not only share a conserved surface epitope with hnRNP K but that it also forms a complex with hnRNP K. Most importantly, hnRNP K, when co-expressed with EBNA2, strongly increases the ability of EBNA2 to activate the viral LMP2A promoter.

**Figure 4:**
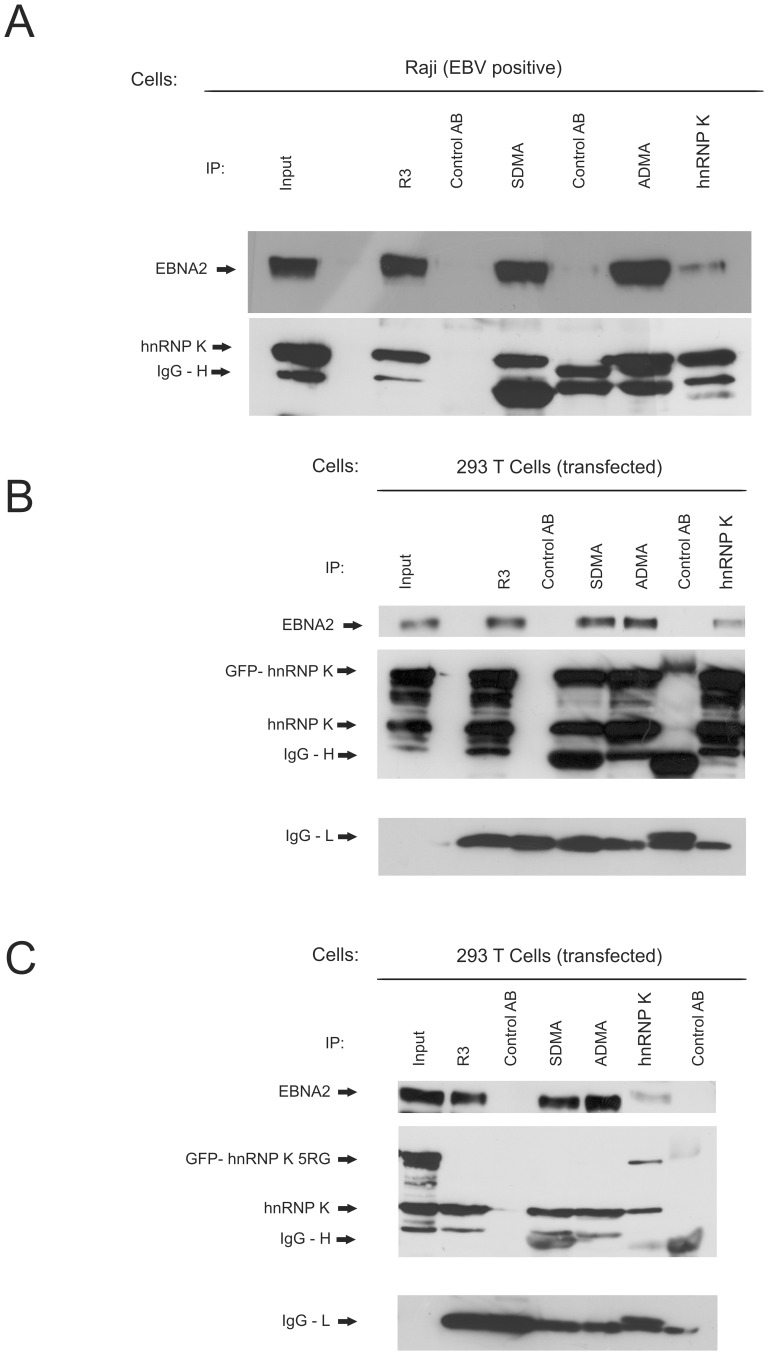
EBNA2 is co-precipitated with wild- type hnRNP K but not with the methylation deficient hnRNP K 5RG mutant. (A) Co-immunoprecipitation of EBNA2 and hnRNP K from EBV positive Raji cells. Raji cells expressing EBNA2 were precipitated with monoclonal antibodies directed against the SDMA- and ADMA- containing Arginine-Glycine (RG)-repeat of EBNA2, an EBNA2 specific mAB (R3) and an hnRNP K specific mAB (D6). The positions of EBNA2 and hnRNP K are indicated by arrows. (B) Co-immunoprecipitation of EBNA2 and GFP - hnRNP K from transfected 293T cells. The cells were precipitated with monoclonal antibodies directed against the SDMA- and ADMA- containing Arginine-Glycine (RG)-repeat of EBNA2, an EBNA2 specific mAB (R3) and an hnRNP K specific mAB (D6). The positions of EBNA2 and hnRNP K are indicated by arrows. (C) No co-immunoprecipitation of EBNA2 and GFP - hnRNP K 5RG is observed from transfected 293T cells. The cells were precipitated with monoclonal antibodies directed against the SDMA- and ADMA- containing Arginine-Glycine (RG)-repeat of EBNA2, an EBNA2 specific mAB (R3) and an hnRNP K specific mAB (D6). The positions of EBNA2 and hnRNP K are indicated by arrows.

## Results

### Monoclonal Antibodies Against SDMA- and ADMA-EBNA2 Precipitate Cellular Proteins with Methylated Arginine Residues

We employed previously developed monoclonal antibodies directed against ADMA- or SDMA-modified EBNA2 [15] for the precipitation of cellular and/or viral proteins from EBV-infected cells. The precipitated proteins were analysed by mass spectrometry as previously described [36] and are listed in Table 1. Also indicated in Table 1 is the methylation status where known. Among the proteins precipitated by the SDMA-antibody, we identified SmD3 known to be a core component of spliceosomal U snRNPs and a major component of the SMN complex (see, for instance, [37], [38]). SmD3 was precipitated both from EBV-infected and non-infected cells as SmD3 has an RG-containing repeat structure comparable to that of EBNA2 which attaches to the SMN protein [35]. The SMN protein was not found, presumably because the binding to the SDMA-modified protein(s) precluded the reaction with the SDMA-specific antibody. To confirm this result, we generated an expression vector for HA-tagged SmD3. In cell extract containing HA-SmD3, we could precipitate SmD3 with the SDMA- but not the ADMA-specific antibody or the EBNA2- specific R3 antibody. The staining of the precipitated SmD3 with the HA-specific antibody is shown in Figure 2A. In the control experiment depicted in Figure 2B, EBNA2 was precipitated with both methylation-specific antibodies and R3 but not with the isotype control antibodies. Note that due to the use of rat and mouse monoclonal antibodies, not all the heavy chains (“IgG-H”) were stained by the secondary antibodies; however, the light chains (“IgG-L”) were clearly present. These data show that the antibodies specific for the methylated surface of EBNA2 react with epitopes on cellular proteins that interact with the same interaction partners, in this case the SMN protein. We were mainly interested in the analysis of proteins identified with the ADMA-specific antibody because our previous analysis had shown that ADMA-EBNA2 is predominantly present at EBNA2-regulated viral promoters [15]. The proteins reactive with the SDMA-antibody were thus not pursued further.

**Figure 5:**
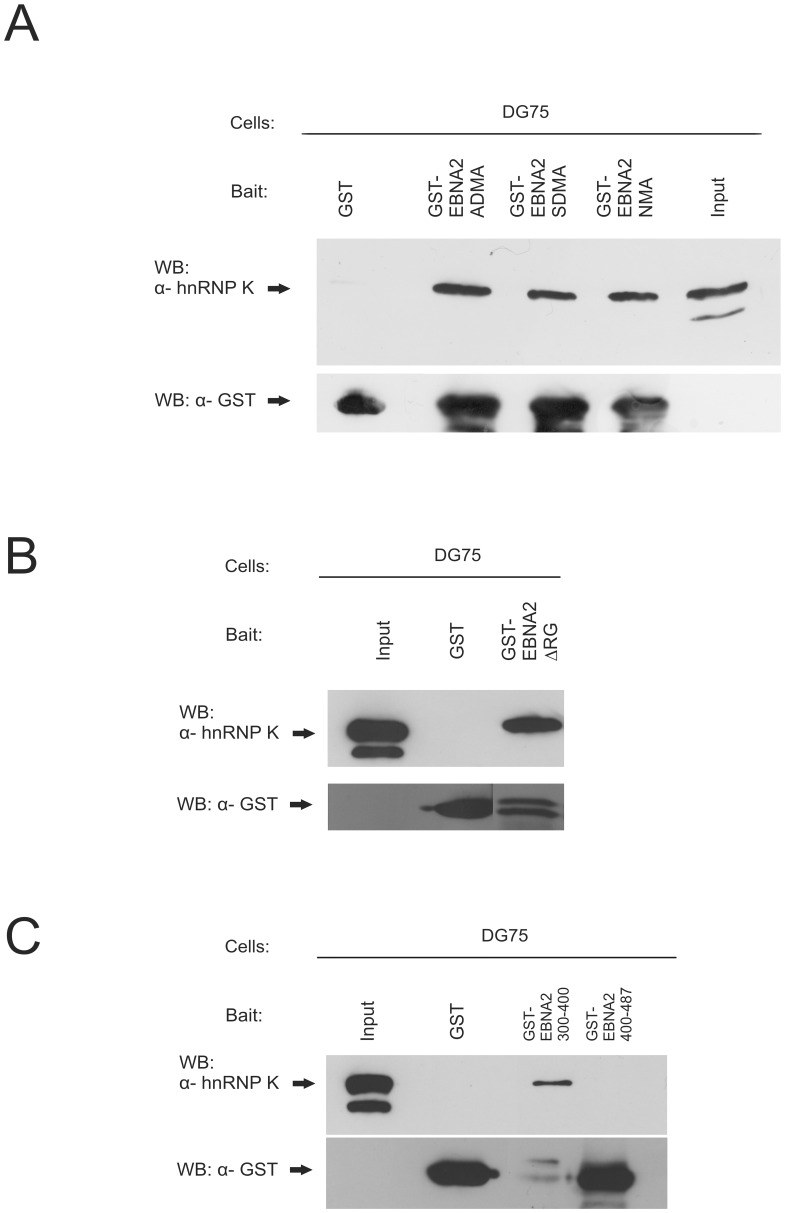
hnRNP K binds to the amino acids 300 – 400 of EBNA2 regardless of the methylation or presence of the RG- repeat. (A) *In vitro* methylated (SDMA and ADMA) and unmethylated (NMA) GST- EBNA2 fusion protein containing amino acids 300–400 of EBNA2 and GST alone were coupled to glutathione sepharose and were incubated with DG75 cell extract treated with methylation inhibitor AdOX. Precipitated hnRNP K was visualised using the hnRNP K mAb D-6. (B) GST- EBNA2ΔRG fusion protein containing amino acids 300–400 without the RG- Repeat of EBNA2 and GST alone were coupled to glutathione sepharose and were incubated with DG75 cell extract. Precipitated hnRNP K was visualised using the hnRNP K mAb D-6. (C) GST- EBNA2 aa400–487 fusion protein containing amino acids 400- 487 of EBNA2, GST- EBNA2 aa300–400 and GST alone were coupled to glutathione sepharose and were incubated with DG75 cell extract. Precipitated hnRNP K was visualised using the hnRNP K mAb D-6.

### EBNA2 Forms a Complex with hnRNP K in EBV-infected Cells

We used the ADMA-specific monoclonal antibodies for the precipitation of methylated proteins from non-infected BL41 and EBV-infected EBNA2-containing Raji Burkitt’s lymphoma cells. Among the proteins identified by the mass spectrometric analysis, we found hnRNP K in both EBNA2-positive and EBNA2-negative cell extracts. To confirm this result, we first subjected extract of non-infected BL41 cells to precipitation using the EBNA2-specific monoclonal antibody R3 which binds at the C-terminus outside of the methylation region [39], the SDMA- and ADMA-antibodies and the hnRNP K specific monoclonal antibody D6. As can be seen in Figure 3A, only the ADMA- or the hnRNP K-specific antibody D6, but not the SDMA-specific antibody precipitated hnRNP K. As expected, R3 did not yield a signal, and the absence of EBNA2 in the BL41 extract was confirmed by western blot (data not shown).

**Figure 6:**
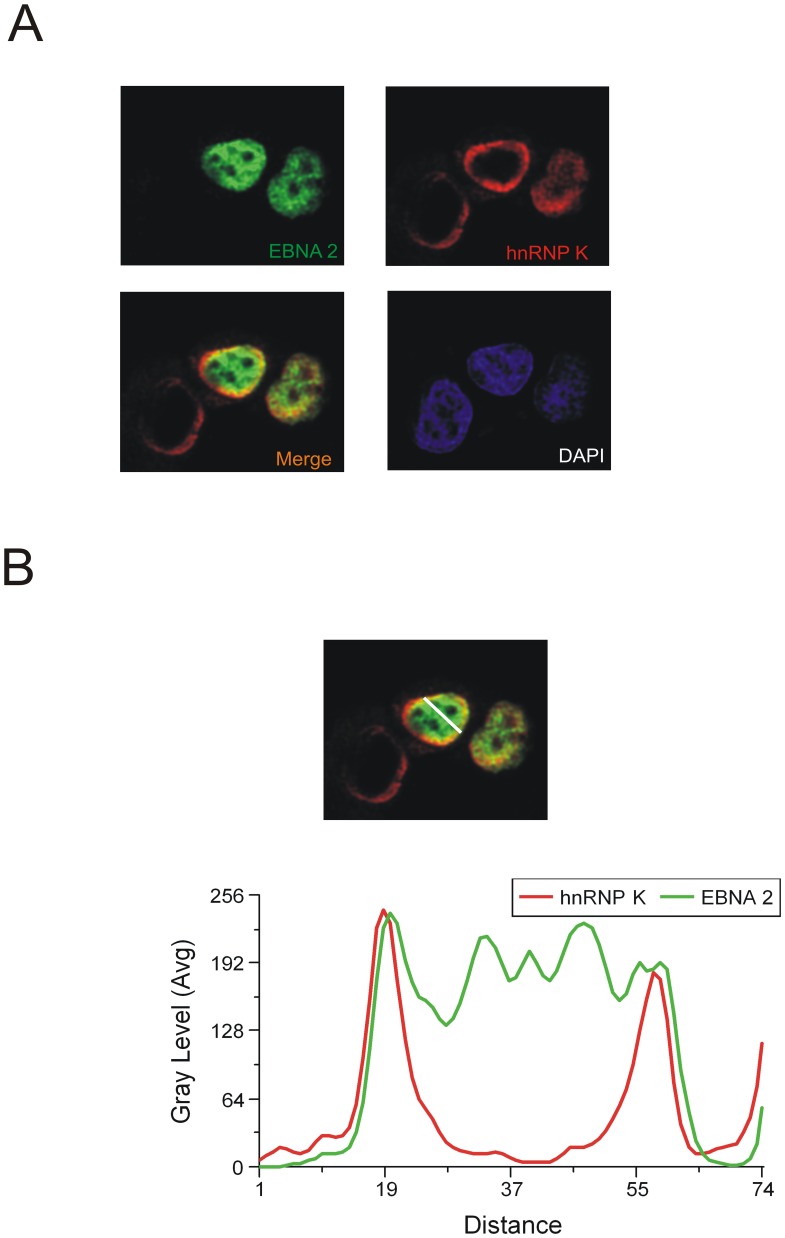
hnRNP K and EBNA2 co-localize in transiently transfected cells. (A) HeLa cells transfected with EGFP- EBNA2 were analysed by confocal laser scanning microscopy. Endogenous hnRNP K was detected using the monoclonal D-6 antibody and an Alexa 647 coupled anti mouse antibody. The signals for hnRNP K (red) or EBNA2 (green) are shown. The merged signals show co-localisation of hnRNP K and EBNA2, resulting in a yellow color. Also shown is the DAPI staining of DNA. The fluorescence profiles of hnRNP K and EBNA2 (B) at a co-localisation hotspot (indicated by the line, left picture - lower lane) were analysed with the Leica MMAF software. The signals for hnRNP K and EBNA2 show the same progression of intensity at the inner nuclear membrane.

We decided to confirm the reactivity of the ADMA-antibody using hnRNP K synthesised and asymmetrically dimethylated in *E. coli* by coexpressed PRMT1. Soluble *E. coli* extract containing ADMA- methylated hnRNP K was subjected to immunoprecipitation with either an hnRNP K-specific monoclonal antibody, the ADMA-specific antibody or the previously described monoclonal antibody against the non-methylated RG-repeat of EBNA2 (NMA) [15]. As shown in Figure 3B, the hnRNP K-specific as well as the ADMA-specific antibody clearly precipitated hnRNP K from the *E. coli* extract confirming the reactivity of the ADMA-specific antibody with hnRNP K in the absence of other eukaryotic proteins. Furthermore, we excluded binding to PRMT1 which is complexed to hnRNP K [29], [40] (data not shown). We also observed binding of a small fraction of hnRNP K by the NMA-antibody. Our previous experiments had shown that this antibody only reacted with (non-methylated) EBNA2 when the EBV-infected cell was treated with the methylation inhibitor Adox [15]. The binding of this antibody to hnRNP K again indicated that non-methylated hnRNP K apparently has a surface at its methylation site similar to the one of EBNA2.

**Figure 7:**
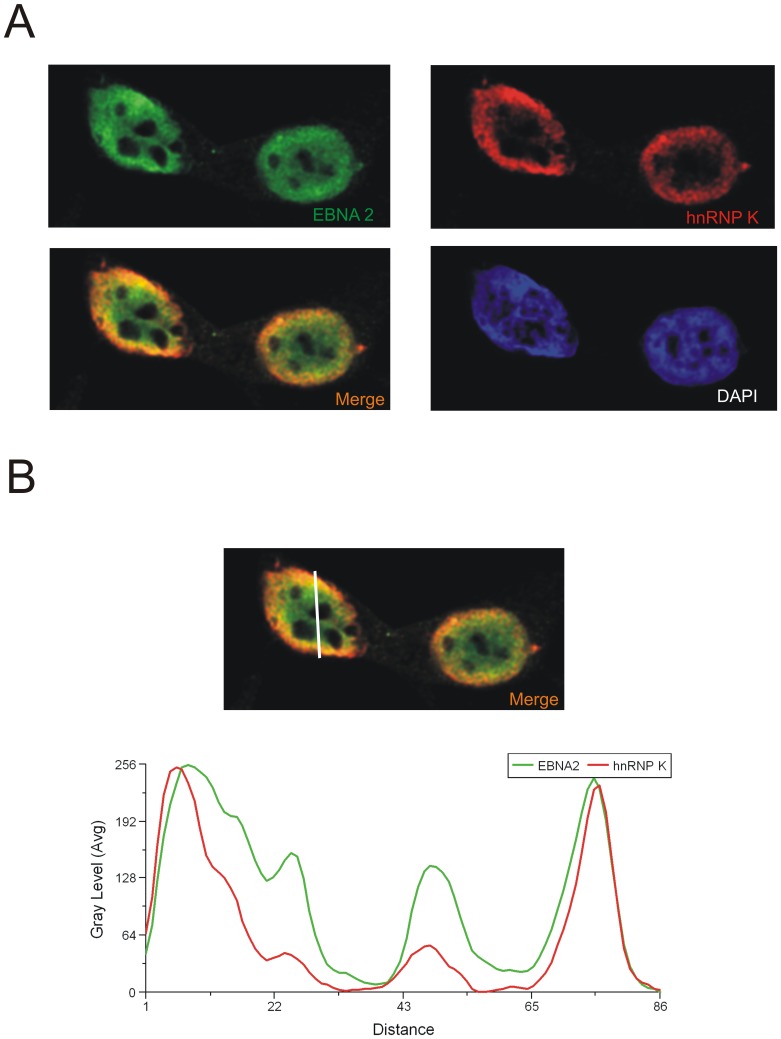
hnRNP K and EBNA2 co-localize in EBV positive cells. (A) 293-EBV cells were analysed by confocal laser scanning microscopy. Endogenous hnRNP K was detected using the monoclonal D-6 antibody and an Alexa 647 coupled anti mouse antibody. Endogenous EBNA2 expressed from the viral episome was detected using the monoclonal R3 antibody and an TRITC coupled anti rat antibody. The signals for hnRNP K (red) or EBNA2 (green) are shown. The merged signals show co-localisation of hnRNP K and EBNA2, resulting in a yellow color. Also shown is the DAPI staining of DNA. The fluorescence profiles of hnRNP K and EBNA2 (B) at a co-localization hotspot (indicated by the line, left picture - lower lane) were analysed with the Leica MMAF software. The signals for hnRNP K and EBNA2 show the same progression of intensity at the inner nuclear membrane.

We next subjected Raji cells to precipitation either with the ADMA-, SDMA- or the methylation-independent EBNA2-specific monoclonal antibody R3, and the hnRNP K specific monoclonal antibody D6. As can be seen in Figure 4A, upper panel, the methylation-specific antibodies and R3 precipitated EBNA2, while the control antibody did not. When the same extracts were probed with the hnRNP K specific antibody D6, hnRNP K was found co-precipitated by R3, the SDMA- and ADMA as well as the hnRNP K antibody (Figure 4A, lower panel). Conversely, the hnRNP K-specific antibody D6 co-precipitated EBNA2. The fact that R3 and also the SDMA-antibody precipitated hnRNP K from the EBNA2-containing extract indicated that both proteins are in a complex with each other, because only EBNA2 but not hnRNP K contains SDMA residues [29].

**Figure 8:**
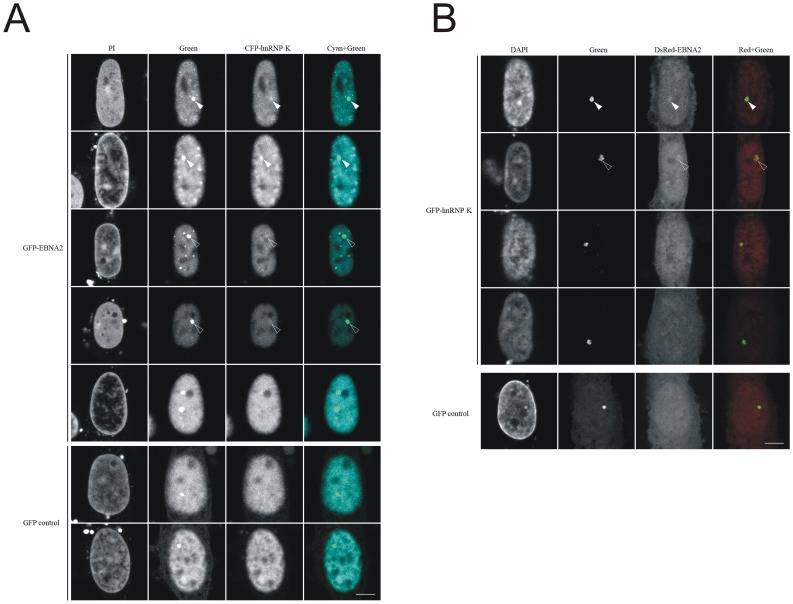
hnRNP K interacts with EBNA2 in a cell based interaction system. Cells containing a *lac* operator (lacO) array inserted in the genome were transfected with expression vectors for a lac repressor fused with a GFP binding protein (GBP) and the indicated fluorescent fusion proteins as indicated. For comparison and orientation the nucleus was stained with PI or DAPI. The GFP fusion proteins are captured at the *lac* operator array by the LacI-GBP and the co-localization of cyan or red fusion proteins visualized. Clear and weak interactions at *lacO* array spots are marked with filled and open arrow tips, respectively. The displayed cells represent the different patterns observed in several independent experiments. GFP expression vectors were used as negative control. Scale bar is 5 µm.

### The EBNA2-hnRNP K-interaction is Dependent of Methylation of hnRNP K

To further investigate if the binding of the two proteins is dependent of the methylation status of hnRNP K, we carried out co- immunoprecipitations from 293T cells either transfected with wild-type GFP- hnRNP K and EBNA2 or the methylation deficient mutant GFP- hnRNP K 5RG and EBNA2. As can be seen in Figure 4B, the binding of EBNA2 to hnRNP K is unaffected by the GFP- tag of hnRNP K and EBNA2 is co- precipitated by the hnRNP K specific antibody and *vice versa*. These findings confirm the results from EBV positive Raji cells (Figure 4A). In contrast, the methylation deficient 5RG mutant of hnRNP K is unable to bind to EBNA2 whereas the binding of EBNA2 to endogenous hnRNP K is unaffected. Furthermore the ADMA- specific antibody is not able to precipitate GFP - hnRNP K 5RG, which further highlights its specificity (Figure 4C).

**Figure 9:**
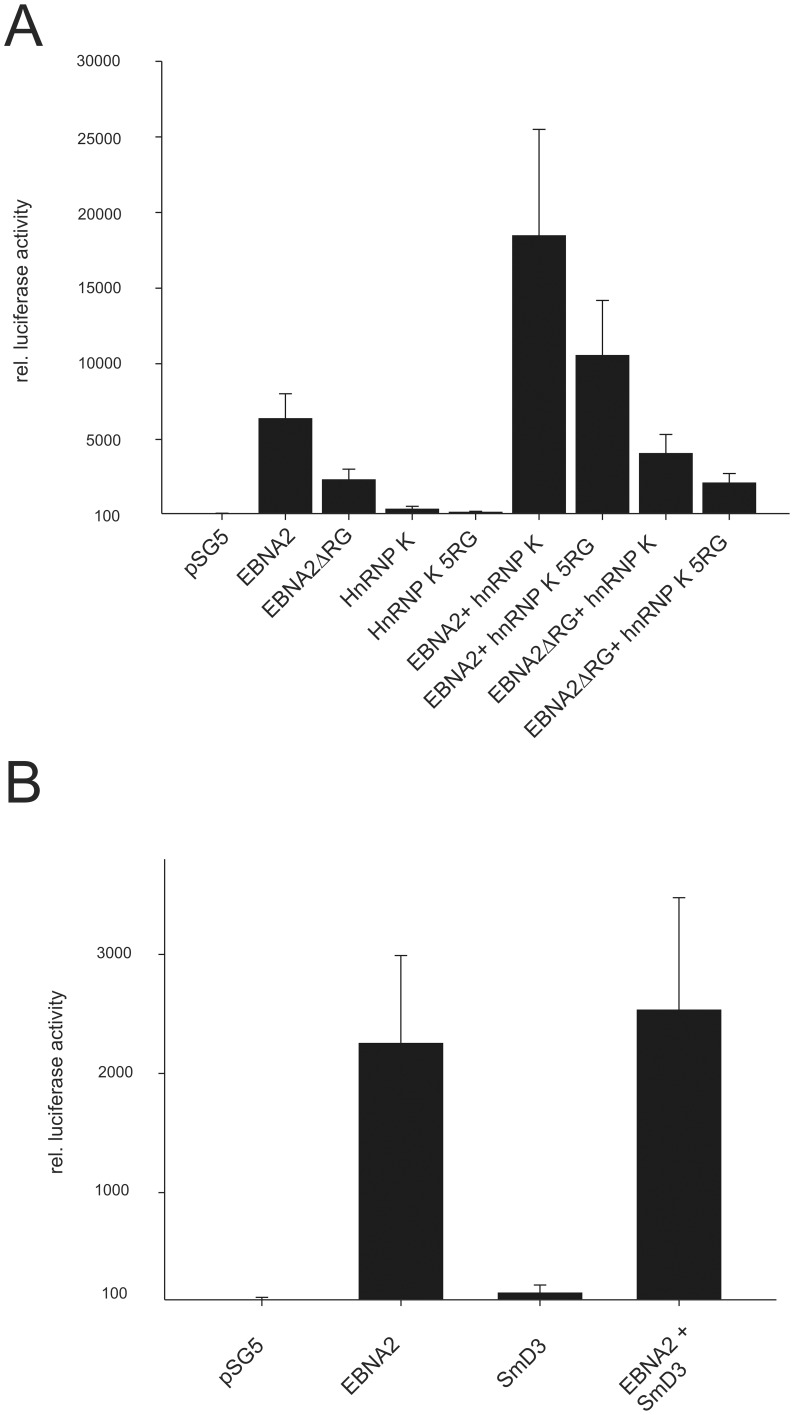
hnRNP K but not SmD3 enhances the EBNA2-mediated promoter activation at the viral LMP2a promoter. (A) A LMP2a promoter luciferase construct was co- transfected into DG75 cells with EBNA2, EBNA2-ΔRG, hnRNP K, and hnRNP K-5RG expression constructs in the indicated combinations. The luciferase value (RLU) obtained with empty pSG5 and the reporter construct was set to 100%. The graph represents the values obtained from 5 independent experiments carried out in duplicate. (B) A LMP2a promoter luciferase construct was co- transfected into DG75 cells with EBNA2 and SmD3 expression constructs in the indicated combinations. The luciferase value (RLU) obtained with empty pSG5 and the reporter construct was set to 100%. The graph represents the values obtained from 4 independent experiments carried out in duplicate.

To corroborate the above results, GST-pull-down assays were carried out. For this purpose, we generated a GST-EBNA2 fusion protein containing aa 300–400 of EBNA2 including the methylation site at 339–354. This fusion protein was then treated either with *E. coli*-expressed His- tagged PRMT1 to generate ADMA-EBNA2 or *Baculovirus* expressed PRMT5/WD45 to generate SDMA-EBNA2. The reaction products were then precipitated with the antibodies to see whether the correct products were formed as the SDMA-specific antibody does not react in a western blot [15]. Treatment with PRMT1 yielded a GST-EBNA2 fusion protein that reacted with the NMA and the ADMA but not the SDMA antibody as shown in Figure S2. The detection of *E.coli*-expressed PRMT1 with the novel PRMT1-specific antibody 7D2 (see Materials and Methods) is shown in Figure S1. This antibody was raised against a peptide encompassing amino acids 250–264 of the human PRMT1 which are divergent from all other known PRMTs. The clone 7D2 (Rat IgG2a) reacted only with a single band in a western blot using DG75 cell extract and migrated to the same position as E.coli-expressed PRMT1 (Figure S1). Methylation with the PRMT5/WD45 complex expressed in *Baculovirus*-infected cells, however, yielded a protein that reacted with the three antibodies indicating that the PRMT5 preparation contained a contamination with a type I PRMT that catalysed the generation of ADMA residues. The precipitation from the untreated extract yielded, in addition to a strong band of non-methylated fusion protein, a faint band reactive with ADMA antibody (Figure S2). We then employed the bacterial fusion proteins for a pull-down of hnRNP K protein from extract of non-infected DG75 lymphoma cells. The extract from these cells was treated with AdOx after lysis to prevent additional and unspecific methylation of the GST-fusion proteins. As shown in Figure 5A, we observed binding of hnRNP K to non-methylated as well as methylated GST-EBNA2 fusion protein. In the control experiment using GST-protein alone, no binding was observed. The observation that non-methylated EBNA2 also binds hnRNP K indicated that residues adjacent to the RG-repeat of EBNA2 might also be involved in binding to hnRNP K. As outlined above, the SDMA-specific antibody co-precipitated hnRNP K from EBNA2-containing cell extract but not from non-infected cells showing that hnRNP K interacted both with SDMA- and ADMA-EBNA2 *in vivo*. The same results (Figure 5B) were obtained with a GST- EBNA2 (aa 300–400) mutant lacking the RG repeat (ΔRG). This indicated also that residues adjacent to the RG-repeat are also involved in the interaction with hnRNP K.

**Figure 10:**
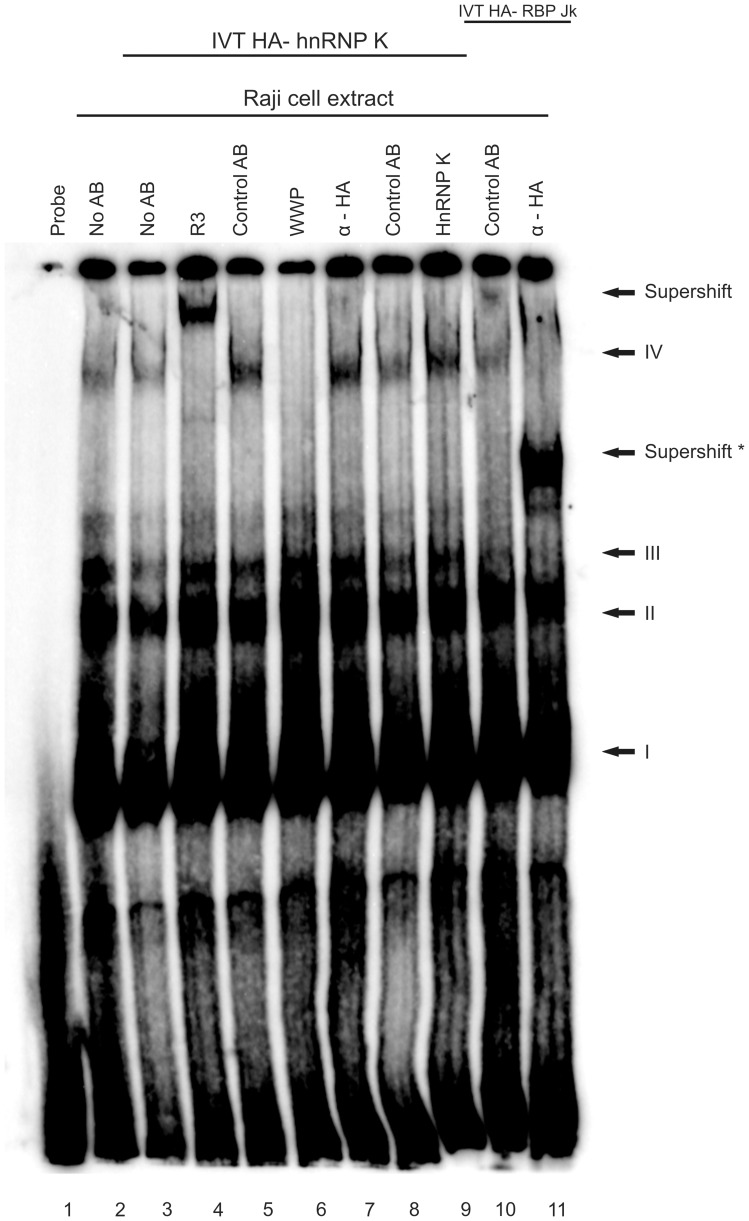
hnRNP K is not present in EBNA2-containing DNA complexes. EBNA2-containing Raji cell extract was incubated with *in vitro* translated hnRNP K and RBP- Jk and antibodies as indicated above and then assayed in a gel shift assay. R3 recognizes EBNA2 regardless of its methylation status and induces a “super- shift” indicated by the upper arrow, the mAb 6C8 directed against the “WWP”-repeat of EBNA2 destroys the EBNA2/RBPjκ-complex IV. Control antibodies corresponded to the respective IgG-subtype of each antibody. To efficiently separate the high molecular weight complexes, the electrophoresis was carried out for an extended time. Therefore, uncomplexed ^32^P-labelled probe ran out of the gel. The position of the RBPjκ-containing complexes I-IV as described in the text are indicated; the arrow (“Supershift”) points at the EBNA2-containing complex IV that is supershifted by R3 but not by the hnRNP K specific D6 antibody or the HA- specific antibody. The arrow (“Supershift *”) indicates the RBP- Jk containing complex which is supershifted by the HA- specific antibody and served as an internal control.

To further characterise the binding of EBNA2 to hnRNP K, a GST- tagged EBNA2 fragment containing the C-terminal amino acids 400–487 were created. As can be seen in Figure 5C, the fragment containing the aa 400–487 is not able to bind hnRNP K in contrast to the EBNA2 fragment consisting of aa 300–400 encompassing the RG-repeat. These results suggest that hnRNP K interacts with EBNA2 via its amino acids 300–400 regardless of the presence of the methylated RG- repeat. A similar observation was previously made for the interaction of the SMN protein with the SDMA-modified RG-repeat of EBNA2, where the main but not exclusive binding region for SMN on EBNA2 was located at and around the RG-repeat [10]. In contrast, the methylation of hnRNP K appears to be necessary for binding to EBNA2 (see also below).

### EBNA2 and hnRNP K Co-localise

To show that EBNA2 also forms a complex with hnRNP K in intact cells, we carried out a co-localisation study using confocal laser scanning microscopy as described previously [32]. For this purpose, EGFP-EBNA2 [32] was expressed in HeLa cells. hnRNP K was visualised using the D6 antibody and secondary Alexa 647 -labelled goat anti-mouse IgG. Co-localisation of EBNA2 with hnRNP K was observed in 35.7% of the cells that expressed both proteins (a representative image is shown in Figure 6). hnRNP K and EBNA2 showed clear co- localisation at numerous spots along the inner nuclear membrane. A representative image of the distribution of fluorescence intensity across a line through the nucleus (“linescan”) is pictured in Figure 6B. The linescan shows that the intensities for the EBNA2 and hnRNP K signals overlap and further supports the notion that the two proteins interact.

The same results were obtained for endogenous proteins using the 293 EBV cell line. The endogenous (i.e. non-transfected) EBNA2 was visualised by the EBNA2- specific R3 antibody and goat- anti- rat TRITC -labelled antibody. hnRNP K was visualised using the D6 antibody and secondary Alexa 647 -labelled goat anti-mouse IgG. A representative cell is pictured in Figure 7A, a linescan showing the same intensity in fluorescence is shown in Figure 7B. These results, in conjunction with the GST-pull-down study and the co-immunoprecipitation experiments (see above), strongly suggest that ADMA- and SDMA-modified EBNA2 and ADMA-methylated hnRNP K form (a) functional unit(s) in EBV-infected cells.

### hnRNP K Interacts with EBNA2 in vivo in a Subset of Cells

To further investigate this interaction we used a cell based protein interaction assay. We immunocaptured the GFP-EBNA2 fusion protein (bait) with the GBP- lacI at the chromosomal lacO array, that becomes visible as distinct nuclear spot (Figure 8A). In about one third of all transfected cells we observed a clear co-localisation of the CFP-hnRNP K fusion protein (prey) at the lacO spot which is indicative of a direct or indirect protein interaction. Another third showed a weak interaction and the remaining cells did not show any clearly detectable interaction and were indistinguishable from GFP control cells. We also performed the reciprocal experiments by switching fluorescent proteins and immobilizing GFP-hnRNP K at the lacO array spot (Figure 8B). In this combination we also observed co-localisation of the DsRed-EBNA2 at the lacO spot although with generally weaker signals. These cell based interaction assay results provide further evidence for an interaction of hnRNP K with EBNA2 *in vivo*. The displayed cells give a representative overview of the observed variability and indicate that this interaction does not occur in all cells or at least not to an equal extent. These results suggest that the hnRNP K interaction with EBNA2 is not constitutive but likely subjected to some additional regulation.

### hnRNP K Enhances EBNA2-mediated Activation of the Viral LMP2A Promoter

hnRNP K has repeatedly been shown to directly activate [41], [42], [43], [44] or inactivate [45], [46] transcription. To test whether the interaction of EBNA2 and hnRNP K changed the transactivation of a viral promoter by EBNA2, we co-expressed EBNA2-wt, hnRNP K-wt, the methylation-deficient mutants EBNA2-ΔRG [16] and hnRNP K-5RG [31] in all possible combinations together with a luciferase reporter driven by the promoter of the viral LMP2A [32]. As shown in Figure 9A, EBNA2-wt activated the promoter by about 500-fold (p = 0.0000000016), while the activation by EBNA2-ΔRG was lower but still highly significant (p = 0.000009975). hnRNP K-wt or the 5RG-mutant alone exerted a small but significant activation on the promoter construct (p = 0.000173 and p = 0.00529, respectively), while co-expression of hnRNP K-wt enhanced the EBNA2-wt-mediated activation by up to three-fold (p = 0.001675). The hnRNP K- 5RG mutant co-activated EBNA2-wt to a smaller degree than hnRNP K-wt (p = 0.00833). The activity of EBNA2-ΔRG was only slightly increased by hnRNP K- wt (p = 0.00261), and no co-activation was observed for the combination of both mutants (p = 0.875). We determined the relative levels of EBNA2 in the presence or absence of hnRNP K expression. The co-expression of hnRNP K did not change the EBNA2 level excluding a trivial explanation for the observed effect (Figure S3). This result and the results from interaction analysis using the hnRNP K-5RG mutant (Figure 4C) strongly support the notion that the interaction between EBNA2 and hnRNP K is mainly (but not exclusively) mediated by the methylation of the two proteins. The expression levels of transfected EBNA2-wt, EBNA2-ΔRG, hnRNP K-wt and hnRNP K-5RG are shown in Figure S4. We used the co-expression of SmD3 which was precipitated by the SDMA antibody (Table 1) as an additional negative control. SmD3 which does not bind to EBNA2 was not able to co-activate EBNA2 in this assay as shown in Figure 9B.

### hnRNP K is not Present in EBNA2-DNA Binding Complexes

To see whether hnRNP K is present in EBNA2-DNA complexes, we carried out a gel-shift experiment employing cell extract from EBV/EBNA2-positive Raji cells. As indicated in Figure 10, we added either HA- hnRNP K or HA- RBPjk to the Raji cell extract. EBNA2 binds DNA via the repressor RBPjκ [47], and this interaction can be inhibited by the antibody 6C8 directed against the WWP-repeat of EBNA2 [48] that is used for its interaction with RBPjκ (reviewed in [49]). We have previously shown that the ADMA-form of EBNA2 is preferentially present at EBNA2-regulated promoters [15]. We thus tested whether the hnRNP K- antibody D6 which co-precipitates EBNA2 (see Figure 4A) would induce a super- shift as observed for the EBNA2-specific antibody R3 with a DNA-fragment derived from the viral LMP2A promoter. To exclude a possible unsuitability of the D6 antibody for EMSA we also tested the HA antibody in the samples which included HA- tagged hnRNP K or RBPjk, the latter serving as internal positive control. As shown in Figure 10, the EBNA2/ RBPjκ -containing complex designated “IV” was super-shifted by R3, while the antibody D6 against hnRNP K and the HA- antibody did not. In contrast, the HA- antibody was able to bind to and super-shift the HA- RBPjk bound to DNA (“Supershift*”). As internal control we used the antibody 6C8 which interferes in the EBNA2- RBPjκ interaction [48]. As can be seen, this antibody diminished the signal from complex IV. The absence of hnRNP K in the EBNA2/DNA-complex indicates that the observed co-activation by hnRNP K is not mediated by direct promoter binding of hnRNP K. The interaction of the two sub-forms of EBNA2 (SDMA and ADMA) which differ in their presence in EBNA2- DNA-binding complexes [15] to hnRNP K hints at the possibility that EBNA2 and hnRNP K co-operate in other activities, for instance in post-transcriptional processing of mRNA. The latter possibility will have to be addressed in a different set of experiments beyond the scope of this communication.

## Discussion

The hypothesis underlying our analysis was that EBNA2, as demonstrated for its interaction with RBPjκ *via* its “WWP”-motif, uses the methylated RG-repeat to attach to cellular factors to use or interfere with their functions. For instance, EBNA2 binds with its SDMA- modified RG- repeat to the SMN protein and with the ADMA-modified RG-repeat to the HERV-K (HML-2) NP9 protein [10], [32]. We therefore employed recently developed monoclonal antibodies against the SDMA and ADMA-repeat of EBNA2 for the identification of cellular proteins with a similar surface structure. Of the proteins precipitated by the SDMA-EBNA2-specific antibodies, we notably detected SmD3 known to be SDMA-modified by PRMT5 [33], [34] and PRMT7 [50]. The precipitation of SmD3 strengthened our hypothesis that the antibody recognised not only the methylated arginine residues of EBNA2 but also the tertiary structure of the RG-motif. The RG-motif confers binding of both the SDMA-SmD3 and the SDMA-EBNA2 to SMN [10], [35]. Importantly, the ADMA-EBNA2-antibody, in contrast to the SDMA-antibody, did not bind SmD3 as it does not contain ADMA residues [51]. Because SmD3 is a common component of spliceosomal U snRNP it was not surprising that the SDMA-specific antibody also precipitated components of the spliceosome, namely the U1 snRNP-specific 70K protein as well as the U5 snRNP component PRPF8 [52]. Since it was likely that these proteins co-precipitated with SmD3 due to being part of the same RNP complexes we did not pursue the SDMA-precipitated proteins further.

Of the proteins identified by the ADMA-specific antibodies, nothing is known about the Ras-GTPase-activating protein SH3-domain-binding protein variant (gi: 62896771) identified in our study. Its splice variant, G3BP1, was shown to be associated with SMN and with Caprin-1 which was formerly known as GPI-anchored membrane protein 1 or cell cycle associated protein [53], [54]. Caprin-1 was also detected in our analysis. The role of Caprin-1 and the G3BP1 variant in EBV transcription or replication is unknown, however, a role of Caprin-1 in *Vaccinia* virus replication was previously demonstrated [55]. The G3BP1 variant contains several RGG and RG motifs at its C-terminus and might thus also be arginine-methylated. The ATP-dependent RNA helicase A (DHX9) contains ADMA and its arginine methylation is a prerequisite for nuclear localisation [56]. Like hnRNP K, DHX9 was previously found to be associated with the EBV-encoded nuclear antigen 5 (EBNA5 or EBNA-LP) [57]. As EBNA2 binds to the RNA helicase DDX20 (DP103/Gemin3) [9], it is possible that DHX9 and EBNA2 also form a complex. This is presently being investigated.

hnRNP K is highly conserved in eukaryotic cells and plays a role in various cellular processes like chromatin remodelling, transcriptional regulation, splicing, translation or signal transduction (see, for example, [30], [44], [58], [59], [60], [61]). hnRNP K interacts directly or indirectly with a large number of cellular proteins [62], most notably with proteins involved in RNA metabolism. Because hnRNP K also plays a role in transcriptional activation and since the ADMA-form of EBNA2 is predominantly bound to EBNA2-responsive promoters [15], we decided to analyse the precipitation of hnRNP K by the ADMA-specific antibody in greater detail. As a precedent, the cross-reactivity between an epitope on hnRNP K and the PTB-associated splicing factor was demonstrated recently [63]. Through the use of bacterial expressed hnRNP K, which contained exclusively ADMA- methylated arginine residues we could clearly show that the ADMA-specific antibody binds to methylated hnRNP K. Interestingly, an antibody directed against non-methylated EBNA2 also detected hnRNP K indicating that both proteins share a common surface structure that is most likely used for the interaction with cellular partner proteins. Most importantly, hnRNP K and EBNA2 bind to each other, presumably *via* the methylated regions as protein arginine methylation is used either for protein-RNA or protein-protein interactions [21]. However, the GST-pull-down analysis showed that the non-methylated EBNA2 and the ΔRG mutant also bind to hnRNP K indicating that the region surrounding the RG-repeat is also involved in binding. This is in line with the previously described association of EBNA2 with SMN, where the binding to SMN is mainly but not exclusively mediated via the RG-repeat of EBNA2 [10]. In the living cell, the EBNA2-hnRNP K-interaction might be regulated *via* methylation or another secondary modification, as we observed an interaction in the lacO-based assay system only in about 60% of the cells that expressed the GFP-labelled proteins (Figure 6). While EBNA2 does not exist in non-methylated form [15], it is possible that newly synthesized hnRNP K might undergo cell cycle –dependent differences in methylation. The functional significance of the EBNA2-hnRNP K-interaction was emphasised by the observation that hnRNP K enhanced the EBNA2-mediated activation of the viral LMP2A promoter by more than 3-fold. Interestingly, the activation of the viral C promoter by the hnRNP protein AUF1 was described by Ling and co-workers; however, the interaction domains between EBNA2 and AUF1 were not mapped [64].

A previous report showed that hnRNP K is present in transcriptionally active sites in EBV-transformed cells and that hnRNP K was highly enriched at loci with high EBV viral RNA content [65]. This is reflected by the fact that hnRNP K strongly co-activated EBNA2. The observation that the splicing machinery was distributed randomly *vis-à-vis* the viral DNA but was enriched at the transcript site [65] indicated that there is a recruitment of splicing factors to nascent viral transcripts. The role of EBNA2 in this process remains unclear. However, we assume that the interaction between EBNA2 and hnRNP K indicates a co-operation during transcription and that the binding of EBNA2 to proteins of the splicing machinery reflects the close link between transcription and splicing [66], [67]. However, the lack of hnRNP K at EBNA2- containing DNA complexes indicates that the enhancement of LMP2A expression might take place at a post-transcriptional level. Further studies will be needed to address the question whether the binding of EBNA2 influences other activities of hnRNP K, *i.e.* the known interaction with c- Src or its activity in mRNA translation, *i.e.* the *c-myc* gene, which is a target for both hnRNP K and EBNA2 [43], [68], [69], [70].

## Materials and Methods

### Cell Lines and Tissue Culture

HEK 293-T, 293-EBV and HeLa cells were cultured in DMEM medium (GIBCO), supplemented with 10% FCS and antibiotics, non-adherent cell lines were grown in RPMI 1640 medium (GIBCO), supplemented with 10% FCS, Na-Pyruvate and antibiotics. The EBV-infected cell lines Raji and 293-EBV, the EBV- negative cell lines DG75 [71] and BL- 41 as well as 293-T and HeLa cells were previously described [32], [72].

### Transfection/Electroporation/Luciferase Assay

For transient expression of the various proteins, 5×10^6^ 293-T cells were transfected with 8 µg/10 cm dish of the expression vectors and combinations thereof using Nanofectin® (PAA, Cölbe, Germany). Western blotting by the ECL^®^-method (GE Healthcare, Munich, Germany) was carried out as described. Electroporation and luciferase analysis was carried out as described [32]. DG75 cells were electroporated using a Bio-Rad Gene Pulser at 250 V and 950 µF. Briefly, 10^7^ cells were washed once and resuspended in 0.25 ml of ice-cold RPMI 1640 without supplements and placed on ice. Then, 4 µg of reporter plasmid, 10 µg of each respective effector plasmid, and 2 µg of peGFP-C1 (Clontech, Palo Alto, CA, USA) were added. Parental pSG5 vector (Agilent Technologies, Waldbronn, Germany) was used to adjust DNA amounts. After electroporation, cells were kept on ice for 10 min, suspended in 10 ml of RPMI with 20% fetal calf serum, and grown for 48 h. To determine the transfection efficiency, 100 µl of the cells were fixed and analysed in a Becton Dickinson FACScan analyser for eGFP-positive cells, gated on the living population. The remainder of cells were washed in PBS and lysed in 100 µl 1× CCLR-buffer (Promega, Mannheim, Germany). The luciferase activity of the supernatants was determined in a Lumat LB9501 (Berthold, Bad Wildbad, Germany) by using the Promega luciferase assay system® (Promega) as recommended by the manufacturer.

### Plasmids

To express quantitatively asymmetrically methylated hnRNP K *in E. coli* the plasmid pET28-PRMT1, a kind gift of X. Cheng, Emory University, Atlanta, GA, [73] was completed with a second Shine-Dalgarno- and PRMT1 coding sequence (PCR primers 5′ AAACTCGA G AACTTTAAGAAGGAGATATACCATG 3′; 5′ TTTCTCGAGTTCAGCGCATCCGGTA GTCGG 3′) inserted into *Xho I* and a Shine-Dalgarno- (His_6_)-hnRNP K sequence [74] (PCR primers 5′ TTTGTCGACAAC TTTAAGAAGGAGATATACCATG 3′; 5′ TTTGTCGACCCGGATCATCAGTGGTG 3′) in the *Sal I* site. peGFP- EBNA2 was described previously [11]. pSG5 –HA - hnRNP K and pSG5- HA- hnRNP K 5RG were constructed using the peGFP- hnRNP K and peGFP- hnRNP K 5RG plasmids [29]. dsRed- EBNA2 was constructed using the peGFP- EBNA2 plasmid [11] and the dsRed Monomer C1 vector (Clontech). peCFP- hnRNP K was constructed using the peGFP- hnRNP K plasmid [29] and the peCFP- C1 vector (Clontech). GST- EBNA2 fragment fusion proteins were constructed using the pGEX- 4T1 Vector (Amersham). The complete coding sequence of PRMT1 was amplified by PCR from a HeLa-cDNA library with primers 5′PRMT1-TACAGGATCC *ATG*GAGGTGTCCTG TGGCCAGGCG G-3′ and 3′PRMT1 5′-GACGGGATCCGAATTCAGCGCATCCGGTAGTCGGTGGAGCAG -3′ and cloned into the BamHI-digested eukaryotic expression vectors pSG5 or the BamHI-digested pGEX-4T1 vector for expression of a GST-PRMT1 fusion protein in *E.coli*.

### Preparation of Native Whole Cell Extract

Raji or DG75 cells were lysed for 30 min on ice in PBS supplemented with 0.5% IGEPAL (Sigma) and 0.15 M NaCl and protease inhibitors (Complete mini®, Roche). The lysate was centrifuged at 15,000×g for 15 min, and the supernatant was used for further analysis.

### Antibodies

The rat mAb 8C3 (IgG2b) reacts with NMA-EBNA2, the mouse mAb 13B10 (IgG2c) recognises SDMA-EBNA2, the mouse mAb 6F12 (IgG2b) binds to ADMA-EBNA2 [15], and the rat mAb R3 (IgG2a) binds to a C-terminal epitope outside the methylation region of EBNA2 [39]. Monoclonal anti-hnRNP K antibody (D-6) was from Santa Cruz (Heidelberg, Germany), goat- anti- mouse Alexa 647 was from Life Technologies (Invitrogen, Darmstadt, Germany), peroxidase-coupled anti-rat or anti-rabbit IgG were from Sigma (Munich, Germany). The monoclonal antibody 3F10 (Roche, Penzberg, Germany) binds to the HA-tag. For production of anti-PRMT1 monoclonal antibodies, a peptide encompassing amino acids G_250_MRPNAKNNRDL_264_ of human PRMT1 coupled to BSA was used to immunize Lou/C rats according to a standard protocol [75]. A clone designated 7D2 (Rat IgG2a) that reacted with GST-PRMT1 but not an irrelevant GST-fusion protein in a western blot was stably subcloned and used for further analysis. The reactivity of this antibody with *E.coli*-expressed non-fused PRMT1 and GST-PRMT1 as well as endogenous cellular PRMT1 from the human cell line DG75 [71] is shown in Figure S2.

### Confocal Immunofluorescence Microscopy

HeLa cells were seeded on microscopy cover slips. Cells were transfected with a plasmid encoding EGFP-EBNA2 [32] and endogenous hnRNP K was visualized with the D6 antibody and secondary Alexa 647-labeled goat anti-mouse IgG2a (Invitrogen, Molecular Probes). Nuclei were stained with DAPI. Slides were mounted using Vectashield (Vector Laboratories). Fluorescence images (Figure 6) were captured with a laser scanning microscope, Leica TCS SP2 (Leica Microsystems, Heidelberg, Germany) equipped with an HCX PL APO 63×1.40 NA oil immersion objective lens using scan settings of pinhole 1.0 Airy units, 512×512 pixel image format, four frame averages, and a TD488/543/633 dichromatic beam splitter. Fluorescence spill-over was excluded by using sequential image recording and tightly controlled excitation power and detection channel settings (EGFP-EBNA2 excitation: 44% of 488-nm laser; Alexa 647 excitation: 81% of 633-nm laser, DAPI excitation: 49% of 405-nm laser). The co-localisation of endogenous (i.e. non-transfected) EBNA2 and hnRNP K was carried out in 293-EBV cells [76]. EBNA2 expressed from the viral episome was detected using the monoclonal R3 antibody and a TRITC-coupled anti-rat antibody. Endogenous hnRNP K was visualized with the D6 antibody and secondary Alexa 647-labeled goat anti-mouse IgG2a. Secondary antibodies were highly cross-adsorbed and showed not cross-recognition. Images were captured using the TCS SP5 II/AOBS Leica confocal system (Figure 7). Fluorescence images were acquired in a sequential scan mode with HyD detectors with tightly controlled laser powers and acquisition windows to prevent spill-over (scan 1∶4% 405-nm with 3% 561-nm; scan 2∶6% 488-nm with 16% 633-nm). All images were recorded as stacked series of confocal single z-planes (step size: 488 nm using magnification with 4× frame average of 630× with zoom factor of at least 2.5. Editing of contrast and brightness was applied to the whole image using Leica LAS AF software. For EBNA2-hnRNP K co-localisation, 56 double-positive cells expressing both fusion proteins were evaluated. Co-localisation was analysed using the Leica Lite software profile tool. Co-localisation hotspots were defined as regions with coinciding high fluorescence intensity of hnRNP K and EBNA2 in the same optical z-plane. The percentage of cells showing co-localisation was calculated among the cells expressing both proteins. Additional de-convolution was performed using the Autoquant plug-in of the Leica MMAF Software (Leica Microsystems, Heidelberg, Germany).

### Cell Based Protein Interaction Assay

Fluorescent two-hybrid assays [77] were performed with a few modifications to visualize and test protein interactions. BHK cells containing a *lac* operator repeat array inserted in the genome [78] were seeded on coverslips and cultured in DMEM medium with 10% FCS. After attachment cells were co-transfected with expression vectors for the indicated fluorescent fusion proteins and a GBP- LacI fusion [79] using polyethylenimine (Sigma). After about 16 h cells were fixed with 3.7% formaldehyde in PBS for 10 min, washed with PBST (PBS with 0.02% Tween), stained with DAPI or PI and mounted in Vectashield medium (Vector Laboratories) (Figure 8). For PI staining RNA was eliminated by RNase treatment after fixation.

### Immunoprecipitation

The rat monoclonal antibody (mAb) R3 (rat IgG2a) recognises a C-terminal epitope of EBNA2 while the clone 6C8 (rat IgG2a) binds to the Trp-Trp-Pro motif of EBNA2 and interferes with binding to RBPjκ [48]. For immunoprecipitation appropriate mouse or rat IgG isotype controls were used. For precipitation, 400 µl of mAb supernatant were coupled to 100 µl of settled protein-G-sepharose (PGS, GE Healthcare, München, Germany) for 1 h at 4°C under agitation, sedimented at 5.000 rpm and washed once with 1 ml of lysis buffer 1. For precipitation experiments either 400 µg protein of native whole cell extract or 100 µg protein of native nuclear extract was added and incubated for 2 h at 4°C under agitation, washed three times with lysis buffer 2 (PBS with 0.5% IGEPAL and 0.5 M NaCl) and once with lysis buffer 1. The pellet was resuspended in 2× SDS sample-buffer and incubated for 10 min at RT or heated at 98°C.

### Co-immunoprecipitation Analysis

Raji cells were lysed for 30 min on ice in PBS supplemented with 0.5% IGEPAL (Sigma) and 0.15 M NaCl containing protease inhibitors (Complete mini^®^, Roche, Penzberg, Germany). After incubation, the solution was sonicated with a 10 s pulse and centrifuged at 13.000×g for 15 min, and the supernatant was used for further incubation with antibody immobilised on 30 µl of settled protein G Sepharose^®^ (GE Healthcare). The cells were washed twice with ice-cold PBS and lysed for 30 min on ice in buffer 1 (PBS supplemented with 0.5% IGEPAL (Sigma) and 0.15 M NaCl) containing protease inhibitors (Complete mini®, Roche). After incubation, the solution was sonicated with a 10 s pulse, centrifuged at 13,000×g and 4°C and incubated for 4 h at 4°C with antibody immobilised on 30 µl of settled protein G sepharose (GE Healthcare). The beads were collected and washed repeatedly with lysis buffer. The immune complexes were dissolved in SDS-gel buffer and separated in 10% polyacrylamide gel electrophoresis and transferred to a nitrocellulose membrane. The antibody R3 binds to the C-terminus of EBNA2. The antibodies 6F12 and 13B10 which recognise the asymmetrically and symmetrically di-methylated arginine-Glycine repeat of EBNA2, respectively, and the GST-specific monoclonal antibody 6G9 have been described recently. hnRNP K was detected using monoclonal antibody D6 (sc-13133, Santa Cruz, Heidelberg, Germany).

### In vitro Methylation Assays

Competent *E. coli* BL21- bacteria were transformed with appropriate expression vectors, protein expression was induced with IPTG and soluble extracts were purified with NAP™25 columns (GE Healthcare, Freiburg, Germany) as described previously [32]. *In vitro* methylation assays were carried out using 20 µl of His-PRMT1 extract or 5 µl of PRMT5/WD45 extract, 20 µl of GST- EBNA2 (aa300-400) fusion protein extract and 5 µl of 0.5 M SAM. The mixture was incubated for 1 h at 37°C.

*GST- pull- down assays:* For GST- EBNA2 and GST competent *E. coli* BL21- bacteria were transformed and protein expression was induced with IPTG as described previously [32]. The GST fusion proteins were adsorbed to glutathione-Sepharose beads (GE Healthcare, Freiburg, Germany) for 2 h at 4°C with rotation, and subsequently washed twice with lysis buffer containing 0.15 M NaCl. For binding of cellular proteins to the GST fusion proteins, typically 500 µl of AdOX- treated native whole cell extract (see above) was added to the mixture and incubated for 2 h at 4°C with rotation and subsequently washed 5 times with lysis buffer containing 0.5 M NaCl. The beads were suspended in SDS-gel electrophoresis buffer, boiled and separated by 10% SDS-PAGE. GST-EBNA2 and GST were detected in western-blotting using 6G9 antibody. hnRNP K was detected using the D-6 monoclonal antibody (Santa Cruz , D-6, sc-28380).

### Electrophoretic Mobility Shift Assay (EMSA)

Nuclear cell extracts were prepared essentially as described [80]. Shortly, approx. 10^8^ cells were collected for 5 minutes at 1200 rpm and 4°C and washed twice with cold PBS. The pellet was resuspended in a 4-fold volume of buffer A (10 mM HEPES pH 7.9, 10 mM KCl, 1.5 mM MgCl_2_) [80] and kept on ice for at least 20 minutes. Cells were broken up by several strokes in a dounce homogenizer until the lysate contained about 50% intact nuclei by staining with Trypan blue. The lysate was centrifuged for 15 seconds at 14.000 rpm and 4°C and washed once with a 2-fold volume of buffer A. The resulting nuclear pellet was resuspended a 1-fold volume of buffer B (20 mM HEPES pH 7.9, 420 mM NaCl, 1.5 mM MgCl_2_, 2 mM EDTA pH 8.5) and kept on ice for 30 minutes. The lysate was centrifuged for 20 minutes at 15.000 rpm and 4°C and the supernatant was used for further experiments or stored at –80°C. Electrophoretic mobility shift assays were carried out exactly as described [4], [81]. The probe used for EMSA is derived from the viral LMP2a promoter and contains two RBPjκ–binding sites. For supershift experiments, we employed the EBNA2-specific rat monoclonal antibody R3 [39] or an appropriate isotype (rat IgG2a) control. The monoclonal antibody 6C8 binds to the Trp-Trp-Pro (“WWP”) motif of EBNA2 interferes with binding to RBKJκ and destroys the EBNA2-containing DNA-RBPjκ-EBNA2-complex [48]. *In vitro* transcription-translation of HA-tagged hnRNP K and HA-tagged RBPjκ using vector AJ247 [82] was performed using the TNT® Coupled Reticulocyte Lysate System (Promega, Mannheim, Germany) as described [10], [15] following the instruction of the manufacturer. Typically, 50 µl of the transcription-translation mix were programmed with 1 µg of vector DNA using T7 RNA polymerase.

## Supporting Information

Figure S1 **Expression control of His- PRMT1 and characterization of the PRMT1 specific rat monoclonal antibody 7D2.** *E.Coli* extract containing His- tagged PRMT1, *E.Coli* extract containing GST- tagged PRMT1 and DG75 whole cell extract was analysed by western blotting.
(TIF)

Figure S2 **In vitro methylation of GST-EBNA2-300-400.** The *E.coli*-expressed unmethylated GST-EBNA2-300-400 fusion protein was subjected to *in vitro* methylation by PRMT5/WD45 purified from a baculovirus expression system or PRMT1 expressed in *E.coli*. The methylated fusion proteins as well as an unmethylated control were immunoprecipitated with the EBNA2- methylation specific antibodies (NMA, SDMA and ADMA) and the appropriate isotype controls. Precipitated GST-EBNA2-300-400 fusion protein was detected in a western blot using the GST-specific 6G9 monoclonal antibody.
(TIF)

Figure S3 **EBNA2 expression is not affected by hnRNP K.** DG75 cells were transfected with pSG5 – EBNA2 and pSG5-HA- hnRNP K and the cell extract was analysed by western blotting. EBNA2 was visualized using the R3 antibody, hnRNP K was visualized with the D6 antibody.
(TIF)

Figure S4 **Expression control of the plasmids used in luciferase activity assays.** DG75 cells were transfected with pSG5 – EBNA2, pSG5 – EBNA2ΔRG, pSG5-HA- hnRNP K and pSG5-HA- hnRNP K 5RG and the cell extract was analysed by western blotting. EBNA2 was visualized using the R3 antibody, HA- hnRNP K and HA- hnRNP K 5RG was visualized with the HA antibody.
(TIF)
