# Supplementary figures and images for: Binding of the Heterogeneous Ribonucleoprotein K (hnRNP K) to the Epstein-Barr Virus Nuclear Antigen 2 (EBNA2) Enhances Viral LMP2A Expression
Source: PLoS One. 2012 Aug 3;7(8):e42106. doi: 10.1371/journal.pone.0042106 (PMC3411732; doi:10.1371/journal.pone.0042106)

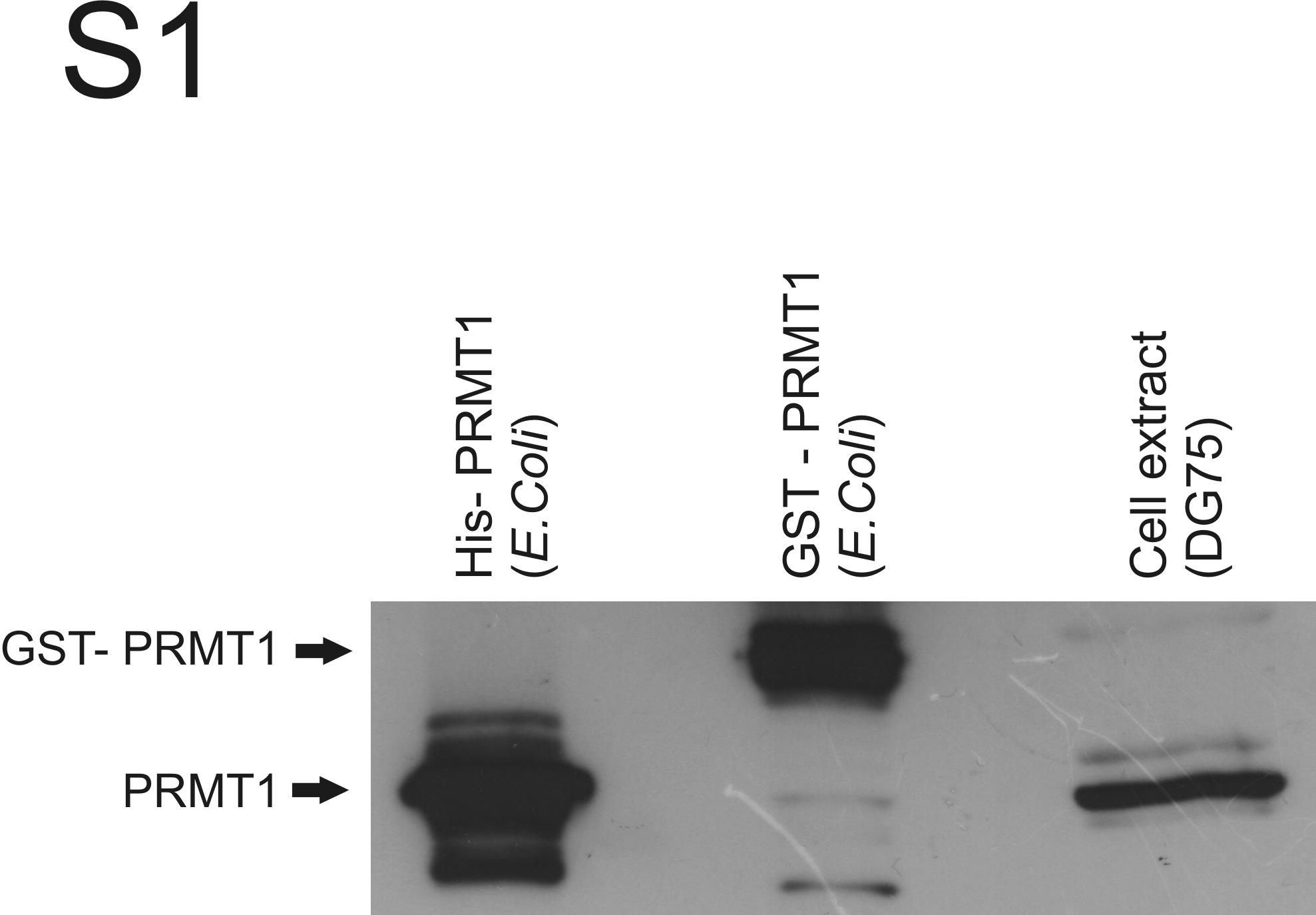

Supplement: Figure S1 — Expression control of His- PRMT1 and characterization of the PRMT1 specific rat monoclonal antibody 7D2. E.Coli extract containing His- tagged PRMT1, E.Coli extract containing GST- tagged PRMT1 and DG75 whole cell extract was analysed by western blotting. (TIF) [file pone.0042106.s001.tif]

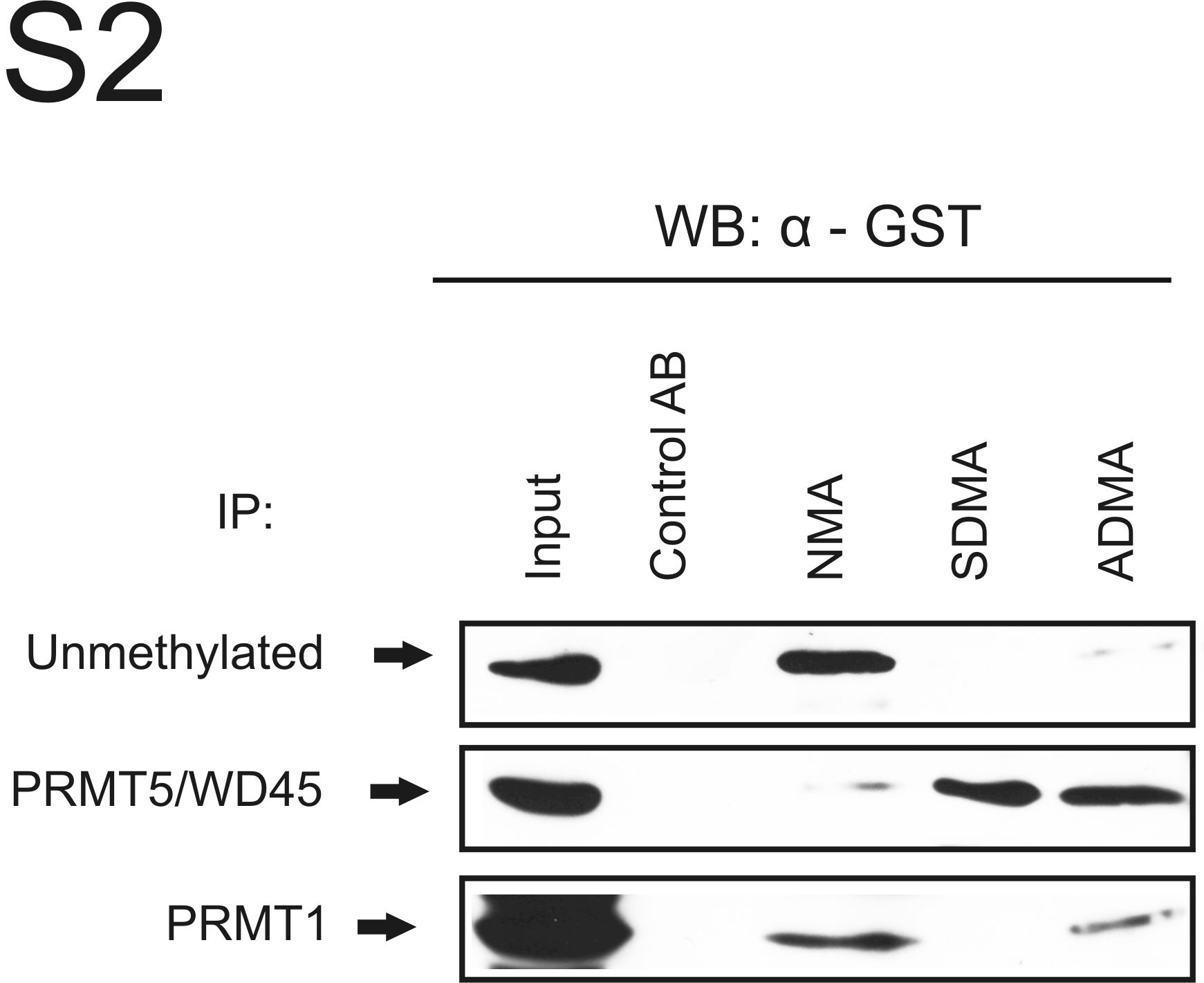

Supplement: Figure S2 — In vitro methylation of GST-EBNA2-300-400. The E.coli-expressed unmethylated GST-EBNA2-300-400 fusion protein was subjected to in vitro methylation by PRMT5/WD45 purified from a baculovirus expression system or PRMT1 expressed in E.coli. The methylated fusion proteins as well as an unmethylated control were immunoprecipitated with the EBNA2- methylation specific antibodies (NMA, SDMA and ADMA) and the appropriate isotype controls. Precipitated GST-EBNA2-300-400 fusion protein was detected in a western blot using the GST-specific 6G9 monoclonal antibody. (TIF) [file pone.0042106.s002.tif]

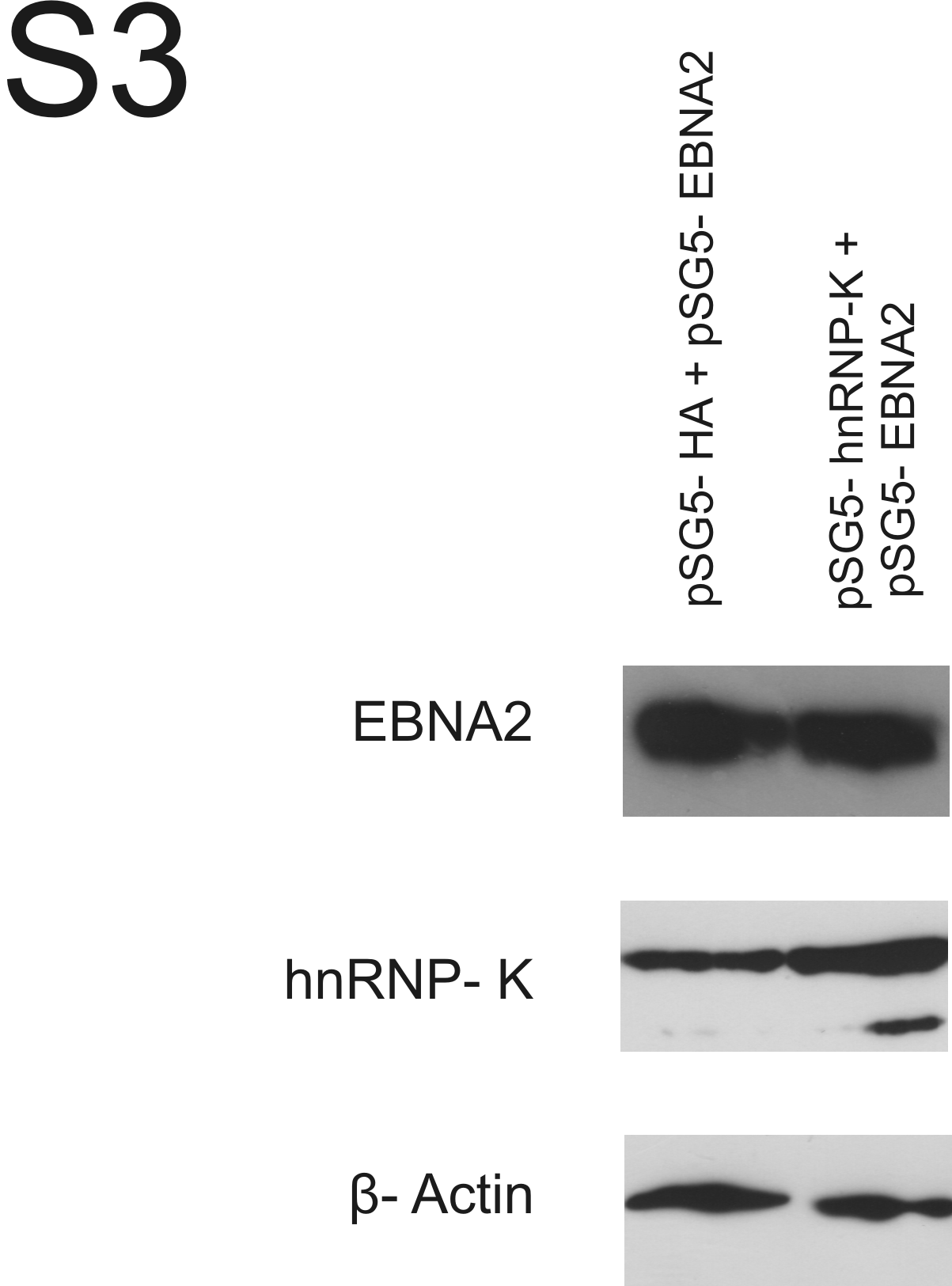

Supplement: Figure S3 — EBNA2 expression is not affected by hnRNP K. DG75 cells were transfected with pSG5 – EBNA2 and pSG5-HA- hnRNP K and the cell extract was analysed by western blotting. EBNA2 was visualized using the R3 antibody, hnRNP K was visualized with the D6 antibody. (TIF) [file pone.0042106.s003.tif]

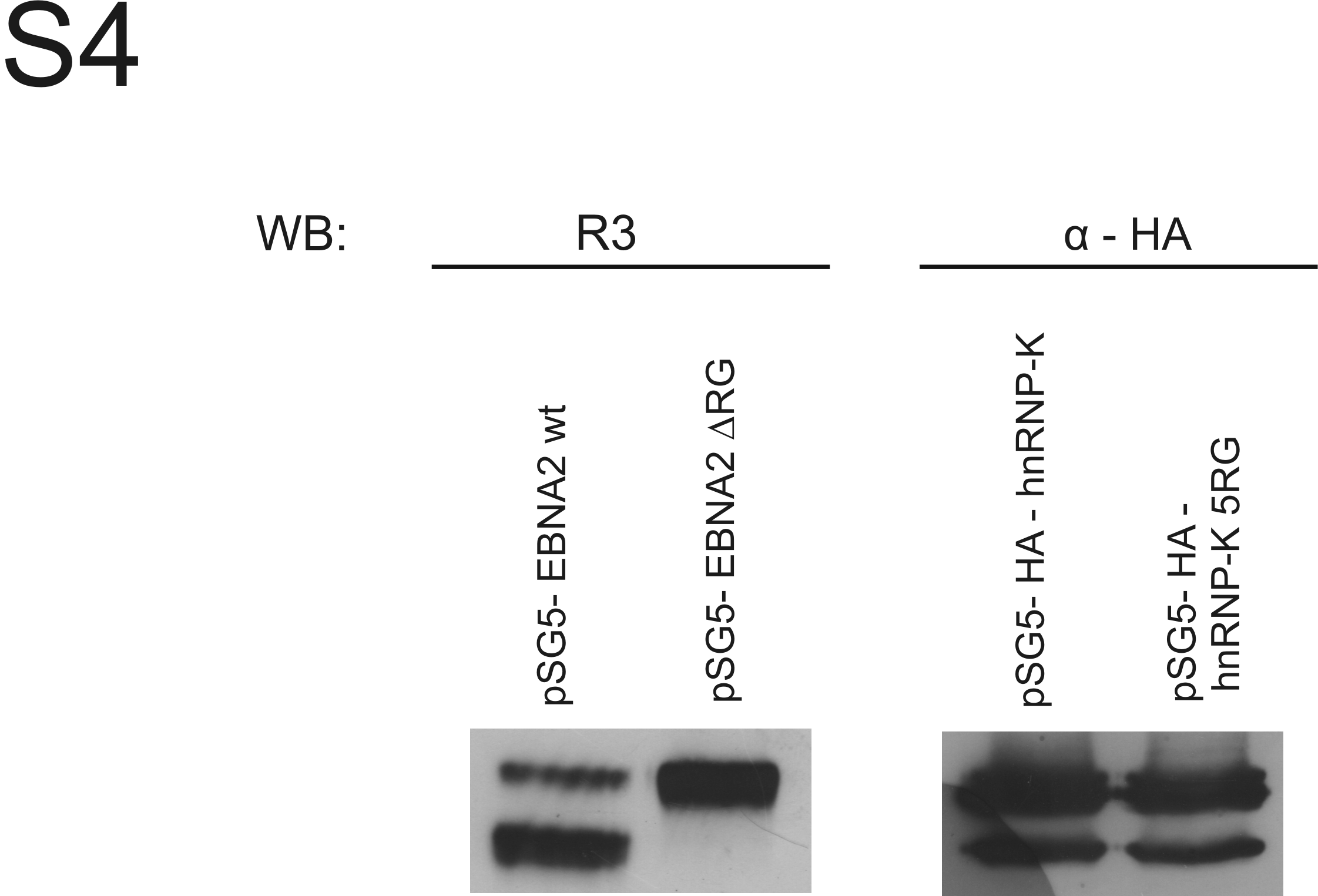

Supplement: Figure S4 — Expression control of the plasmids used in luciferase activity assays. DG75 cells were transfected with pSG5 – EBNA2, pSG5 – EBNA2ΔRG, pSG5-HA- hnRNP K and pSG5-HA- hnRNP K 5RG and the cell extract was analysed by western blotting. EBNA2 was visualized using the R3 antibody, HA- hnRNP K and HA- hnRNP K 5RG was visualized with the HA antibody. (TIF) [file pone.0042106.s004.tif]
